# Correction: Examining relationships between sleep posture, waking spinal symptoms and quality of sleep: A cross sectional study

**DOI:** 10.1371/journal.pone.0306662

**Published:** 2024-07-02

**Authors:** Doug Cary, Angela Jacques, Kathy Briffa

The Results section in the Abstract for this paper is incorrect. The correct Results section is: Compared to Control group participants, those in the Cervical group had more frequent posture changes (mean (SD); 18.3(6.5) versus 23.6(6.6)), spent more time in undesirable/provocative sleep postures (median IQR; 83.8(16.4,105.2) versus 185.1(118.0,251.8)) minutes and had more long periods of immobility in a provocative posture, (median IQR; 0.5(0.0,1.5) versus 2.0(1.5,4.0)). There were no significant differences between the Control and Lumbar groups in the number of posture changes (mean (SD); 18.2(6.5) versus 22.9(9.1)), long periods of immobility in a provocative posture (median IQR; 0.5(0.0, 1.5) versus 1.5(1.5, 3.4)) or the time spent in provocative sleep postures (median IQR; 83.8(16.4,105.2) versus 134.6(40.8, 199.0)) minutes. Participants in both symptomatic groups reported a lower sleep quality than the Control group.

## References

[1] Cary D, Jacques A, Briffa K (2021) Examining relationships between sleep posture, waking spinal symptoms and quality of sleep: A cross sectional study. PLoS ONE 16(11): e0260582. doi: 10.1371/journal.pone.0260582 34847195 PMC8631621

