# Supplementary material for: Examining relationships between sleep posture, waking spinal symptoms and quality of sleep: A cross sectional study
Source: PLoS One. 2021 Nov 30;16(11):e0260582. doi: 10.1371/journal.pone.0260582 (PMC8631621; doi:10.1371/journal.pone.0260582)
Supplement: Attachment — Submitted filename: Response to reviewers.docx [file pone.0260582.s001.docx]

PONE-D-20-35066
Examining relationships between sleep posture, waking spinal symptoms and quality of sleep: A cross sectional study
PLOS ONE

Dear Dr. Cary,

Thank you for submitting your manuscript to PLOS ONE. After careful consideration, we feel that it has merit but does not fully meet PLOS ONE’s publication criteria as it currently stands. Therefore, we invite you to submit a revised version of the manuscript that addresses the points raised during the review process.

Please submit your revised manuscript by Mar 25 2021 11:59PM. If you will need more time than this to complete your revisions, please reply to this message or contact the journal office at [plosone@plos.org](mailto:plosone@plos.org).

When you're ready to submit your revision, log on to <https://www.editorialmanager.com/pone/> and select the 'Submissions Needing Revision' folder to locate your manuscript file.

Please include the following items when submitting your revised manuscript:

- A rebuttal letter that responds to each point raised by the academic editor and reviewer(s). You should upload this letter as a separate file labelled 'Response to Reviewers'.
- A marked-up copy of your manuscript that highlights changes made to the original version. You should upload this as a separate file labelled 'Revised Manuscript with Track Changes'.
- An unmarked version of your revised paper without tracked changes. You should upload this as a separate file labelled 'Manuscript'.

If you would like to make changes to your financial disclosure, please include your updated statement in your cover letter. Guidelines for resubmitting your figure files are available below the reviewer comments at the end of this letter.

If applicable, we recommend that you deposit your laboratory protocols in [protocols.io](http://protocols.io) to enhance the reproducibility of your results. [Protocols.io](http://Protocols.io) assigns your protocol its own identifier (DOI) so that it can be cited independently in the future. For instructions see: <http://journals.plos.org/plosone/s/submission-guidelines#loc-laboratory-protocols>

We look forward to receiving your revised manuscript.

Kind regards,

Matias Noll, Ph.D
Academic Editor
PLOS ONE

## Response to Journal:

# When submitting your revision, we need you to address these additional requirements.

1. Please ensure that your manuscript meets PLOS ONE's style requirements, including those for file naming. The PLOS ONE style templates can be found at

<https://journals.plos.org/plosone/s/file?id=wjVg/PLOSOne_formatting_sample_main_body.pdf> and

<https://journals.plos.org/plosone/s/file?id=ba62/PLOSOne_formatting_sample_title_authors_affiliations.pdf>

***Author’s Response:*** *The above documents have been reviewed. Style requirements have been noted and numerous style changes have been applied to the revised manuscript.*

2. Please clarify whether you have written consent for publication for the participant’s picture in the figure. For further information please refer to our policy on informed consent for publication <https://journals.plos.org/plosone/s/human-subjects-research#loc-Patient-Privacy-and-Informed-Consent-for-Publication>; <https://journals.plos.org/plosone/s/file?id=8ce6/plos-consent-form-english.pdf>

***Author’s Response:*** *The subject has completed and signed the PLOS consent form.*

3. In your Data Availability statement, you have not specified where the minimal data set underlying the results described in your manuscript can be found. PLOS defines a study's minimal data set as the underlying data used to reach the conclusions drawn in the manuscript and any additional data required to replicate the reported study findings in their entirety. All PLOS journals require that the minimal data set be made fully available. For more information about our data policy, please see <http://journals.plos.org/plosone/s/data-availability>.

Upon re-submitting your revised manuscript, please upload your study’s minimal underlying data set as either Supporting Information files or to a stable, public repository and include the relevant URLs, DOIs, or accession numbers within your revised cover letter. For a list of acceptable repositories, please see <http://journals.plos.org/plosone/s/data-availability#loc-recommended-repositories>. Any potentially identifying patient information must be fully anonymized.

Important: If there are ethical or legal restrictions to sharing your data publicly, please explain these restrictions in detail. Please see our guidelines for more information on what we consider unacceptable restrictions to publicly sharing data: <http://journals.plos.org/plosone/s/data-availability#loc-unacceptable-data-access-restrictions>. Note that it is not acceptable for the authors to be the sole named individuals responsible for ensuring data access.

We will update your Data Availability statement to reflect the information you provide in your cover letter.

***Author’s Response:*** *We have reviewed the recommended PLOS documentation and also the original Curtin University Human Research Ethics Committee (HR 140/2014) submission. Our submission and the subsequent approval from the Curtin University Human Research Ethics Committee, only allows for the senior researchers (DC, KB) to access the data. There was no provision provided for public access to the data.*

4. We note that Figure 1 includes an image of a participant in the study.

As per the PLOS ONE policy ([http://journals.plos.org/plosone/s/submission-guidelines#loc-human-subjects-research](about:blank%23loc-human-subjects-research)) on papers that include identifying, or potentially identifying, information, the individual(s) or parent(s)/guardian(s) must be informed of the terms of the PLOS open-access (CC-BY) license and provide specific permission for publication of these details under the terms of this license. Please download the [Consent Form for Publication in a PLOS Journal](about:blank) ([http://journals.plos.org/plosone/s/file?id=8ce6/plos-consent-form-english.pdf](about:blank)). The signed consent form should not be submitted with the manuscript, but should be securely filed in the individual's case notes. Please amend the methods section and ethics statement of the manuscript to explicitly state that the patient/participant has provided consent for publication: “The individual in this manuscript has given written informed consent (as outlined in PLOS consent form) to publish these case details”. If you are unable to obtain consent from the subject of the photograph, you will need to remove the figure and any other textual identifying information or case descriptions for this individual.

***Author’s Response:*** *Consent has been acquired and documented on the PLOS consent form and stored with case details. An additional statement has been included in the revised manuscript line 172-173 to reflect this change.*

## Responses to Reviewers

**Comments to the Author**

1. Is the manuscript technically sound, and do the data support the conclusions?

The manuscript must describe a technically sound piece of scientific research with data that supports the conclusions. Experiments must have been conducted rigorously, with appropriate controls, replication, and sample sizes. The conclusions must be drawn appropriately based on the data presented.

Reviewer #1: Yes

Reviewer #2: Partly

2. Has the statistical analysis been performed appropriately and rigorously?

Reviewer #1: Yes

Reviewer #2: No

3. Have the authors made all data underlying the findings in their manuscript fully available?

The [PLOS Data policy](http://www.plosone.org/static/policies.action#sharing) requires authors to make all data underlying the findings described in their manuscript fully available without restriction, with rare exception (please refer to the Data Availability Statement in the manuscript PDF file). The data should be provided as part of the manuscript or its supporting information, or deposited to a public repository. For example, in addition to summary statistics, the data points behind means, medians and variance measures should be available. If there are restrictions on publicly sharing data—e.g. participant privacy or use of data from a third party—those must be specified.

Reviewer #1: Yes

Reviewer #2: Yes

4. Is the manuscript presented in an intelligible fashion and written in standard English?

PLOS ONE does not copyedit accepted manuscripts, so the language in submitted articles must be clear, correct, and unambiguous. Any typographical or grammatical errors should be corrected at revision, so please note any specific errors here.

Reviewer #1: Yes

Reviewer #2: Yes

5. Review Comments to the Author
Please use the space provided to explain your answers to the questions above. You may also include additional comments for the author, including concerns about dual publication, research ethics, or publication ethics. (Please upload your review as an attachment if it exceeds 20,000 characters).

**Reviewer #1:** The paper presents very interesting results dealing with the interactions between sleep posture and chronic spinal pain with practical clinical perspectives. The variables under study and the experimental design seem to be appropriate.

Nevertheless, I found the abuse of acronyms makes the read of the paper quite difficult.
Please explicit the acronyms more regularly (once by paragraph for example) so that the reader does not have to go back to the beginning to verify the sense of the numerous acronyms used in this paper.

***Author’s Response:*** *Thank you Reviewer #1 for taking the time to provide your valuable suggestions to improve the content and readability of our manuscript. As recommended, we have substantially reduced the number of abbreviations in the manuscript.*

**Reviewer #2:** This paper looks into the association between sleep postures, sleep quality and spinal complaints. Although the study design does not allow a cause and effect analysis, authors suggest that sleep postures affect spinal complaints. From the introduction I understand that both directions of influence are possible though (refs 4, 11-14) .

***Author’s Response****: Thank you Reviewer #2 for your contribution to the improvement of our manuscript. We agree that our findings should only be interpreted as associations and cannot be used to determine cause and effect. This has been stated explicitly in the revised version.*

Here are my comments and suggestions:
Throughout the paper, authors are describing non-significant findings (p>.05) in a confusing way, i.e. using words as ‘more’, ‘poorer’, ‘greater’. This should be avoided.

also when given the exact p value, but especially in the discussion and conclusion where these values do not accompany such statements.

***Author’s Response****: Use of words that described non-significant differences have been removed from the original Abstract, Results and Discussion sections.*

In case authors have reason to belief to be dealing with ‘false negative’ results (type II errors), this should be addressed in the discussion.

***Author’s Response****: The authors believe it possible a Type II error may have occurred when examining differences in the between the Control and Lumbar groups in combined provocative sleep posture and sleep quality. This possibility has been added (line 283), along with the already stated possible influence of recruitment criteria on lines 324 to 329.*

Line 68: suggestion to specify sleep postures. ‘undesirable’ or something likewise?

***Author’s Response:*** *In line 27 we have added the word undesirable to associate it with the phrase provocative sleep postures. At line 60 The words ‘flexion or rotation’ have been added into the text to clarify the applied loads from the related references.*

Line 90: although I am not a native English speaker, ‘greater than’ 46 years old, should be better ‘older than’ or ‘more than’?’

***Author’s Response****: The word ‘older’ has been substituted for ‘greater in line 84. To match the phrasing, the word ‘younger’ has also been substituted for ‘less’ at line 83.*

Lines 103 - 109 : why not link postures videoed with complaints that same morning after. Participants in the asymptomatic group also had occasionally symptoms.

***Author’s Response:*** *Linking morning symptoms with overnight postures is an interesting idea, however our data were not collected with that in mind. Morning data were simply numerical rating scales (NRS) of the severity of pain, stiffness and bothersomeness with no indication of the location or more detailed nature of the symptoms. The morning symptom data were collected to describe the groups in general terms, provide some verification of the group allocations, and to assess whether the nights the sleep data were collected were representative for the participants generally. For the latter, data from the nights of collection were compared to ‘Prior 2 weeks symptoms’ recorded at recruitment*

Line 120: ‘Higher scores indicated increased symptoms for pain, stiffness and bothersomeness and better quality of sleep.’ Is this correct? Lower quality of sleep?

***Author’s Response:*** *That is correct. Higher scores represented a higher quality of sleep. Line 115 has been modified to clarify this relationship.*

Line 151-154: is this sentence grammatically correct?

***Author’s Response:*** *The complete paragraph has been rewritten and simplified.*

Figure 1: suggestion to indicate in this figure how these postures are believed to result in more or less stress on different parts of the spine.

***Author’s Response:*** *The following text has been added after the title* “Sustained postures like rotation, have been identified as causing tissue microdamage and muscle spasms”

Line 171-173: why would the position of the head have no influence on the spinal load (torsion?), considering start of the spine at C1?

***Author’s Response:*** *It is correct that this would create a degree of cervical torsion, however we decided not to focus on this for several reasons.*

1. *We had learnt from our pilot study, that when participants slept in supine, the amount of rotation was < 45 degrees and changed frequently, with participants often rotating their head from left to right several times often only for a few seconds, before becoming static or undertaking a major posture shift.*
2. *The neutral zone of cervical range is considerably larger than the thoracic or lumbar spine, meaning that to reach an end range and cause tissue strain, would most likely require a greater range than 45 degrees.*
3. *Most other researchers only considered supine, prone and side lying in regards to posture and*
4. *We believed the plausible spinal loads would be much larger in association with leg positionings, due to the greater weight of the lower limbs.*

Line 182-184: why didn't you use these data to make groups?

***Author’s Response:*** *Our research questions, groups and the associated exclusion/ inclusion criteria were derived from clinical knowledge. The research design was based on this clinical knowledge and the protocol was registered before data collection commenced. Once the study commenced the registered procedure was followed.*

Line 187: A priori calculated sample size was not reached. This is also less relevant than the power actually obtained with the final dataset. You should report these. See also remark on type 2 errors.

***Author’s Response:*** *The a priori calculated sample size was not reached and this was reported in our updated clinical trials register. This information has now also been added in Lines 193-4. With regards to not finding a significant result comparing the Lumbar and Control groups in regards to sleep posture, we make mention of a possible type II error in line 325.*

Line 216: ‘achieving a normal distribution was difficult’ is a strange way of putting it. Should be rephrased.

***Author’s Response:*** *Thank you for noting this. As the majority of the participants in the control group were symptom free the symptom variables were frequently not normally distributed and attempts to transform the data distribution were rarely successful. The sentence has been rephrased on line 217 and now reads “Achieving a normal distribution was not possible for most Control group variables, particularly patient reported outcome variables, as most participants in this group reported low or no symptom levels”.*

Table 2: suggestion to first explain a and b in the legend.

***Author Response*** *“Data are: ^a^ Mean (Standard Deviation) or ^b^ Median (Interquartile Range)” has been added to the title of Tables 2 and 3.*

Line 302: indeed, this may be. You can use SPSS to see how much power you have, and this should be reported. Also reporting effect sizes may be useful especially when p < 0.05.

***Author’s Response:*** *It is true that post hoc power calculations could be performed, however, inclusion of post-hoc power calculations is controversial and we have elected not to make this change. Zhang et al., (2019)^[1]^ argue that they are conceptually flawed and may be analytically misleading. Moreover, presentation of post-hoc power is complicated by the non-normal distribution of much of our data and the consequent need to use non-parametric statistics. As an alternative approach we have talked about the plausibility of the data in terms of patterns present across multiple variables.*

Line 303: indeed, as suggested above, you can use another approach based on your own observations to allocate into groups.

***Author’s Response****: As noted in our Author’s response above, group characteristics and inclusion criteria were created based on prior clinical experience. The Methodology for this study was reported as part of our clinical trials registration, before we collected any data and we subsequently followed the methodology outlined in the clinical trials registration. We are reluctant to change our methods after examining the data.*

Line 340-351: this info should not be in the conclusion, but rather in the introduction or integrated in the discussion. A conclusion should be concisely answering your research questions.

***Author’s Response:*** *the conclusion has been rewritten (now lines 357 to 363). Extraneous information has been removed and incorporated where appropriate in the Introduction and Discussion sections.*

6. PLOS authors have the option to publish the peer review history of their article ([what does this mean?](https://journals.plos.org/plosone/s/editorial-and-peer-review-process#loc-peer-review-history)). If published, this will include your full peer review and any attached files.
If you choose “no”, your identity will remain anonymous but your review may still be made public.
**Do you want your identity to be public for this peer review?** For information about this choice, including consent withdrawal, please see our [Privacy Policy](https://www.plos.org/privacy-policy).

Reviewer #1: **Yes:** Dr. Olivier Coste (MD,PhD)

Reviewer #2: No.

While revising your submission, please upload your figure files to the Preflight Analysis and Conversion Engine (PACE) digital diagnostic tool, <https://pacev2.apexcovantage.com/>. PACE helps ensure that figures meet PLOS requirements. To use PACE, you must first register as a user. Registration is free. Then, login and navigate to the UPLOAD tab, where you will find detailed instructions on how to use the tool. If you encounter any issues or have any questions when using PACE, please email PLOS at [figures@plos.org](mailto:figures@plos.org). Please note that Supporting Information files do not need this step.

***Author’s Response:*** *The original figure file has been uploaded to PACE and corrected according to the PLOS figure specifications.*

1. Zhang Y, Hedo R, Rivera A, et al. Post hoc power analysis: is it an informative and meaningful analysis? General Psychiatry 2019;32:e100069. doi:10.1136/ gpsych-2019-100069
